# Longitudinal remotely mentored self-performed lung ultrasound surveillance of paucisymptomatic Covid-19 patients at risk of disease progression

**DOI:** 10.1186/s13089-021-00231-9

**Published:** 2021-05-30

**Authors:** Andrew W. Kirkpatrick, Jessica L. McKee, John M. Conly

**Affiliations:** 1 Department of Critical Care Medicine, Foothills Medical Centre, Calgary, Alberta Canada; 2 Department of Surgery, Foothills Medical Centre, Calgary, Alberta Canada; 3 Department of Medicine, Foothills Medical Centre, Calgary, Alberta Canada; 4 Synder Institute for Chronic Diseases, Foothills Medical Centre, Calgary, Alberta Canada; 5 Trauma Program, University of Calgary and Alberta Health Services, Foothills Medical Centre, Calgary, Alberta Canada; 6 Tele-Mentored Ultrasound Supported Medical Interventions (TMUSMI) Research Group, Foothills Medical Centre, Calgary, Alberta Canada

## Abstract

COVID-19 has impacted human life globally and threatens to overwhelm health-care resources. Infection rates are rapidly rising almost everywhere, and new approaches are required to both prevent transmission, but to also monitor and rescue infected and at-risk patients from severe complications. Point-of-care lung ultrasound has received intense attention as a cost-effective technology that can aid early diagnosis, triage, and longitudinal follow-up of lung health. Detecting pleural abnormalities in previously healthy lungs reveal the beginning of lung inflammation eventually requiring mechanical ventilation with sensitivities superior to chest radiographs or oxygen saturation monitoring. Using a paradigm first developed for space-medicine known as Remotely Telementored Self-Performed Ultrasound (RTSPUS), motivated patients with portable smartphone support ultrasound probes can be guided completely remotely by a remote lung imaging expert to longitudinally follow the health of their own lungs. Ultrasound probes can be couriered or even delivered by drone and can be easily sterilized or dedicated to one or a commonly exposed cohort of individuals. Using medical outreach supported by remote vital signs monitoring and lung ultrasound health surveillance would allow clinicians to follow and virtually lay hands upon many at-risk paucisymptomatic patients. Our initial experiences with such patients are presented, and we believe present a paradigm for an evolution in rich home-monitoring of the many patients expected to become infected and who threaten to overwhelm resources if they must all be assessed in person by at-risk care providers.

**Supplementary Information:**

The online version contains supplementary material available at 10.1186/s13089-021-00231-9.

## Introduction

COVID-19 has fundamentally impacted all aspects of human life in 2020. As of December 6 2020, the World Health Organization reported that there had been over 66 million confirmed cases of COVID-19, including 1,523,583 deaths [1], although there may be many more fold cases globally. Globally, various countries and health systems are facing uncertain resource challenges that threaten to overwhelm the capacity to properly care for all citizens. Although very infectious, COVID-19 is very variable in its virulence, being most harmful in older patients, especially those residing in long-term care facilities and having co-morbidities such as hypertension, heart disease, diabetes, and chronic respiratory and kidney diseases [2]. Statistically, young healthy people are still at low risk for progression to severe life-threatening disease and most will experience only mild symptoms and recover by self-isolating at home. However, a small number of such people will deteriorate and become precipitously sick often presenting with advanced pneumonia despite the absence of severe dyspnea due to unique features of the COVID-19 virus. The key to balancing not overwhelming hospital resources, yet still avoiding tragedies, has been stated to employ outreach resources, and being quickly recognizing those few isolated patients who are at risk of deterioration, and “rescuing” them quickly with an aggressive and comprehensive response [3, 4].

The diagnosis of COVID-19 typically relies upon PCR-testing, which unfortunately may have significant false-negative test characteristics. Thus early chest computed tomography (CT) has been recommended as a screening tool for suspected COVID-19 patients with better sensitivity than PCR [5], but this is clearly an impractical solution for home-monitoring. However, interest in using lung ultrasound to diagnose, risk stratify, and manage COVID-19 pneumonia has exploded in the emergency and in-hospital settings, as the distinctive pathological findings of an irregular pleural interface, interstitial “comet-tail” artifacts, and sub-pleural consolidations, are typically peripheral and well seen and quantified by ultrasound [3, 6–9]. An International Panel of Experts recently recommended using tele-ultrasound for remote guidance and consultations in COVID-19 [10]. We fully agree, and further believe that lung ultrasound examinations can be provided to willing self-isolating infected and at-risk patients using informatic technologies to facilitate self-lung ultrasound surveillance [3, 11].

## Remotely telementored self-performed ultrasound (RTSPUS)

Remotely Telementored Self-Performed Ultrasound (RTSPUS) is an innovative technique that was first described as a solution to providing medical imaging onboard the International Space Station, wherein astronauts were guided from earth to image their own bodies under the direction of terrestrial ultrasound experts [12]. We believe that RTSPUS may be an invaluable tool to monitor those at risk of COVID-19 progression while maintaining self-isolation and quarantine, thus preventing any viral transmission and protecting health-care workers and other members of the community. We further believe that almost any anatomical body location that can be reached by a person is amenable to mentored self-ultrasound. In the context of screening for complications of COVID however, infected and at-risk patients are guided to attempt the standardized COVID-19 lung ultrasound examination protocol of Soldati [6]. This examination involves 14 distinct locations (7 per lung; 3 posterior, 2 lateral, and 2 anterior) and is more detailed than many standard examinations that focus solely on the anterolateral chest. As with any ultrasound examination, the exam does not have to be limited to these anatomic locations and may be tailored as a user sees fit. It has also been suggested that the baseline LUS examination includes as many rib spaces as possible so that comparisons can be made as a change from the baseline. Using the RTSPUS technique, the patient is asked to demonstrate the visceral–parietal pleural interface which is viewed and evaluated remotely by the expert clinician, which allows the clinician to detect early deterioration in lung health. For each of the anatomic lung locations, the patient was guided to self-image until the remote mentor was confident that the adequate pleural imaging was captured [13], which included additional imaging with M-mode and Color Power Doppler capturing both the “Seashore [14]” and “Powerslide signs [15]”. While pneumothorax has been reported as a primary complication of COVID-19 [16], they are still rare in a healthy paucisymptomatic population, and these additional signs may not be necessary or practical for widespread adoption of RTSPUS. It should be emphasized that the use of both M-mode and color power Doppler are not typically required to diagnose COVID-19 pneumonia, but were the personal preference of the remote mentor who easily controlled the knobology of the exam remotely.

## Technology

Sterilized ultra-portable economical ultrasound probes interfacing with the patients own smartphones or computing devices can be delivered to patients by any means including commercial couriers and even unmanned aerial vehicles. Thereafter, the informatics can be easily provided using off-the-shelf technologies widely available on a global basis. For example, in our experience, the ultra-portable Philips L12-4 broadband linear array transducer (Philips Company, Eindhoven, Netherlands), attached to a Samsung Galaxy Tablet A (Samsung Corporation, Suwon, Korea), communicating with a remote expert using Zoom Teleconferencing (Zoom, Hillsboro, OH) through a standard smartphone (Samsung Galaxy S20 5G, Samsung Corporation, Suwon, Korea) and with remote mentor control of the ultrasound knobology using the Teamviewer real-time remote access and support software (Teamviewer, Göppingen, Germany), provided a simple off-the-shelf communications package to readily facilitate RTSPUS.

## Patient eligibility

Any conscious motivated patient who is not in severe respiratory distress and able and willing to follow instructions from a remote mentor is a candidate to participate in a remotely guided self-performed home-surveillance lung program. Musculoskeletal limitations of the arms may be a relative contraindication to performing a complete thoracic evaluation of the back, although recent studies have emphasized examinations limited to the anterior chest, with the most relevant areas of surveillance being the most accessible anterior lung segments in a study performed on hospitalized patients [17].

## Current experience

**Fig. 1:**
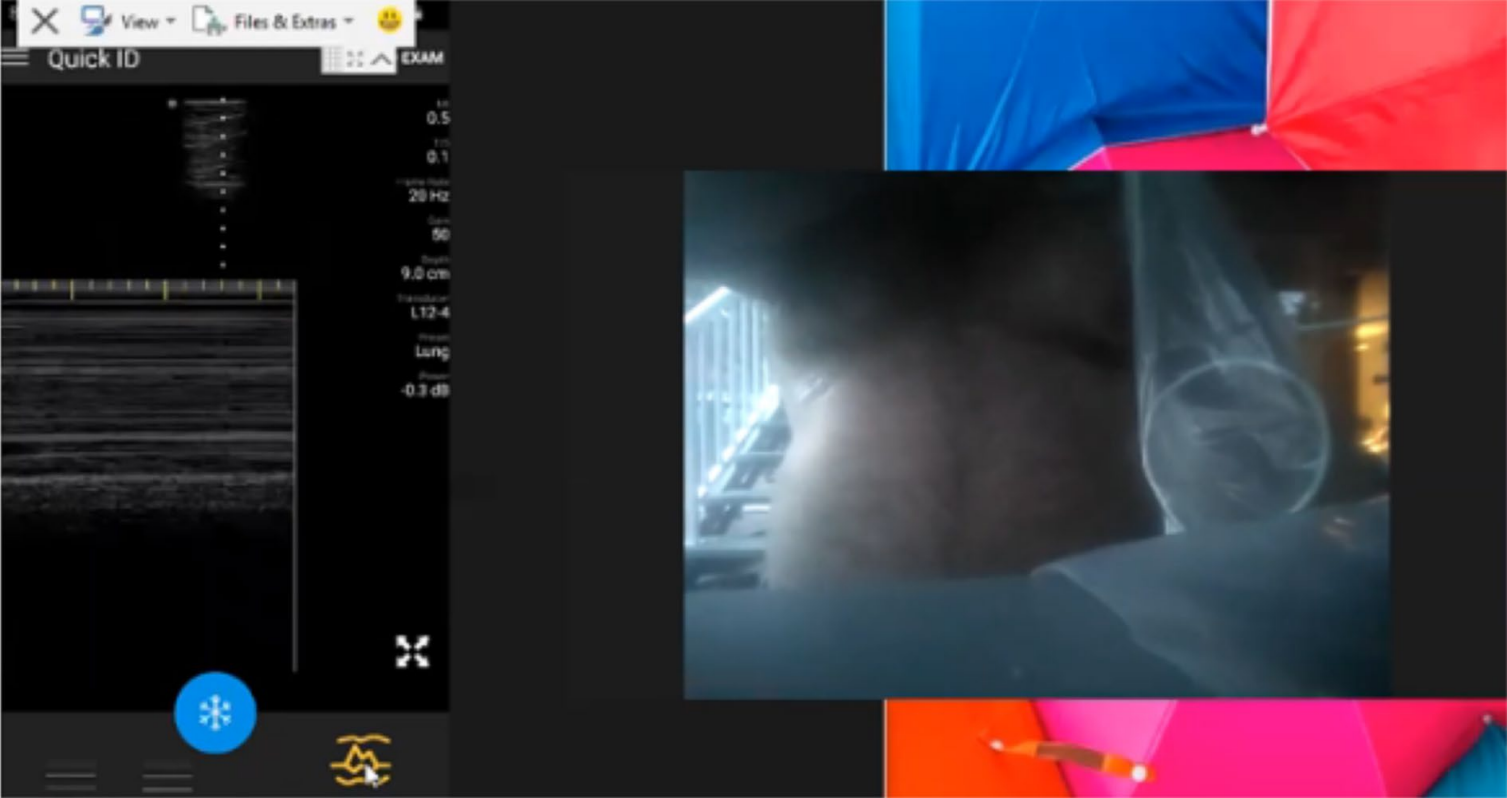
Baseline Self-Performed Lung Ultrasound Study of 32-year-old patient 6 months prior to COVID-19 diagnosis. Screenshot of the Remote Mentors Computer Screen that shows the patient imaging himself to the right of the screen and the resultant ultrasound image showing the “seashore” sign to the left. As lung ultrasound is a dynamic real-time examination hard to capture in any single image, readers are strongly encouraged to view the accompanying supplemental videos of the entire examination online to gain a true appreciation of the technique

**Fig. 2:**
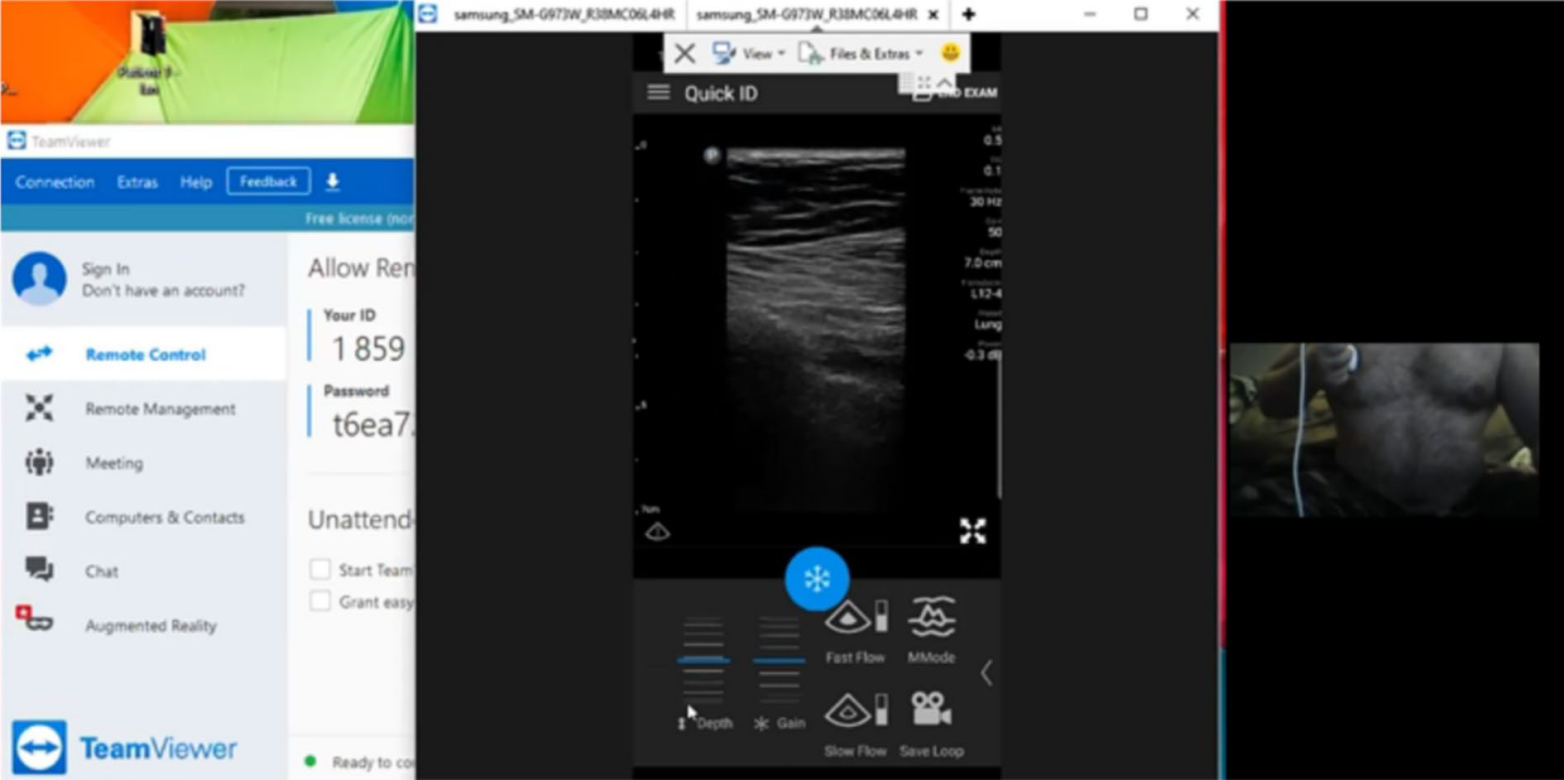
First follow-up Self-Performed Lung Ultrasound Study of 32-year-old patient 2 weeks after COVID-19 diagnosis. Screenshot of the Remote Mentors Computer Screen that shows the patient imaging himself to the right of the screen and the resultant ultrasound image showing the “batwing” sign to the left. As lung ultrasound is a dynamic real-time examination hard to capture in any single image, readers are strongly encouraged to view the accompanying supplemental videos of the entire examination online to gain a true appreciation of the technique

**Fig. 3:**
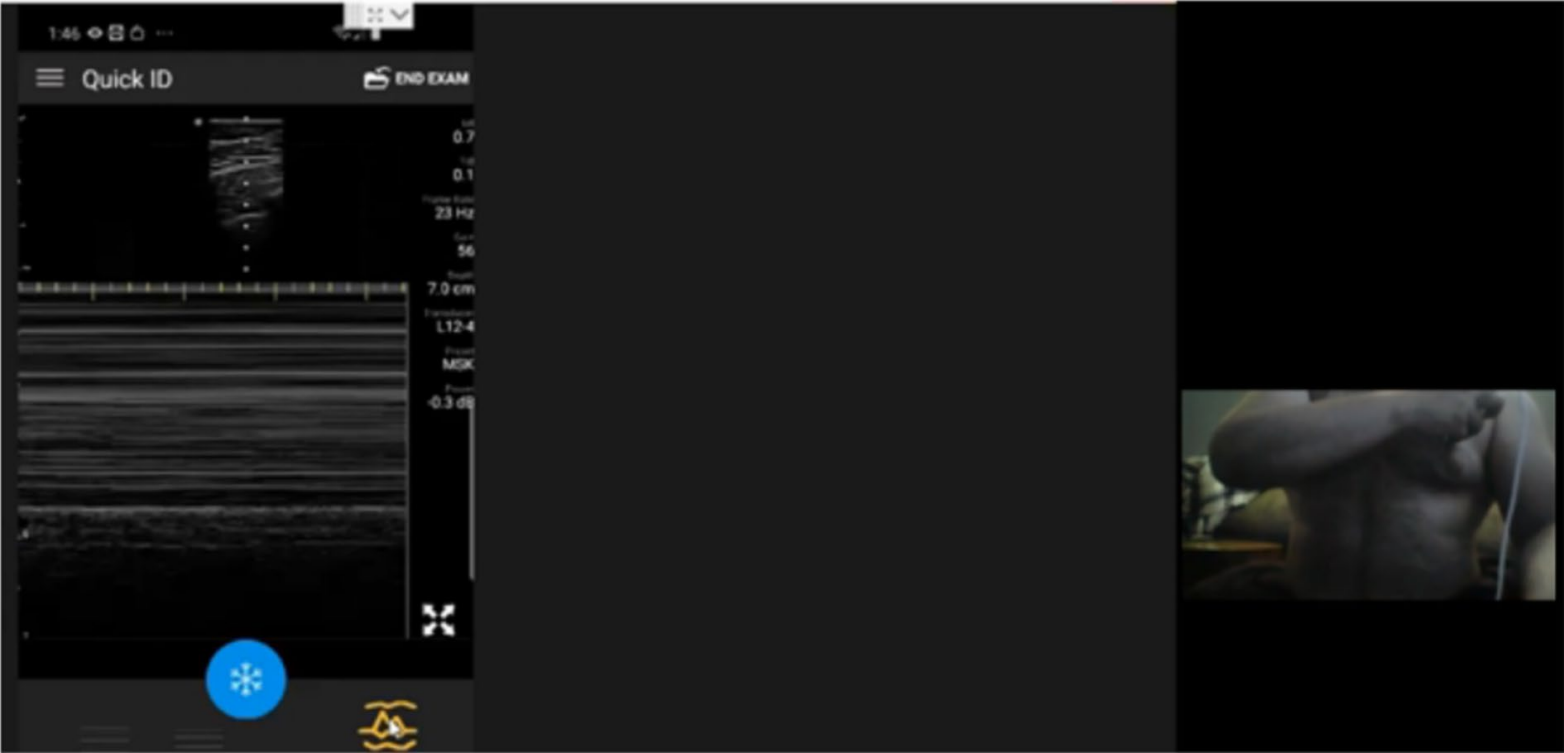
Second follow-up Self-Performed Lung Ultrasound Study of 32-year-old patient 2 weeks 2 days after COVID-19 diagnosis. Screenshot of the Remote Mentors Computer Screen that shows the patient imaging himself to the right of the screen and the resultant ultrasound image showing the “seashore” sign to the left. As lung ultrasound is a dynamic real-time examination hard to capture in any single image, readers are strongly encouraged to view the accompanying supplemental videos of the entire examination online to gain a true appreciation of the technique

**Fig. 4:**
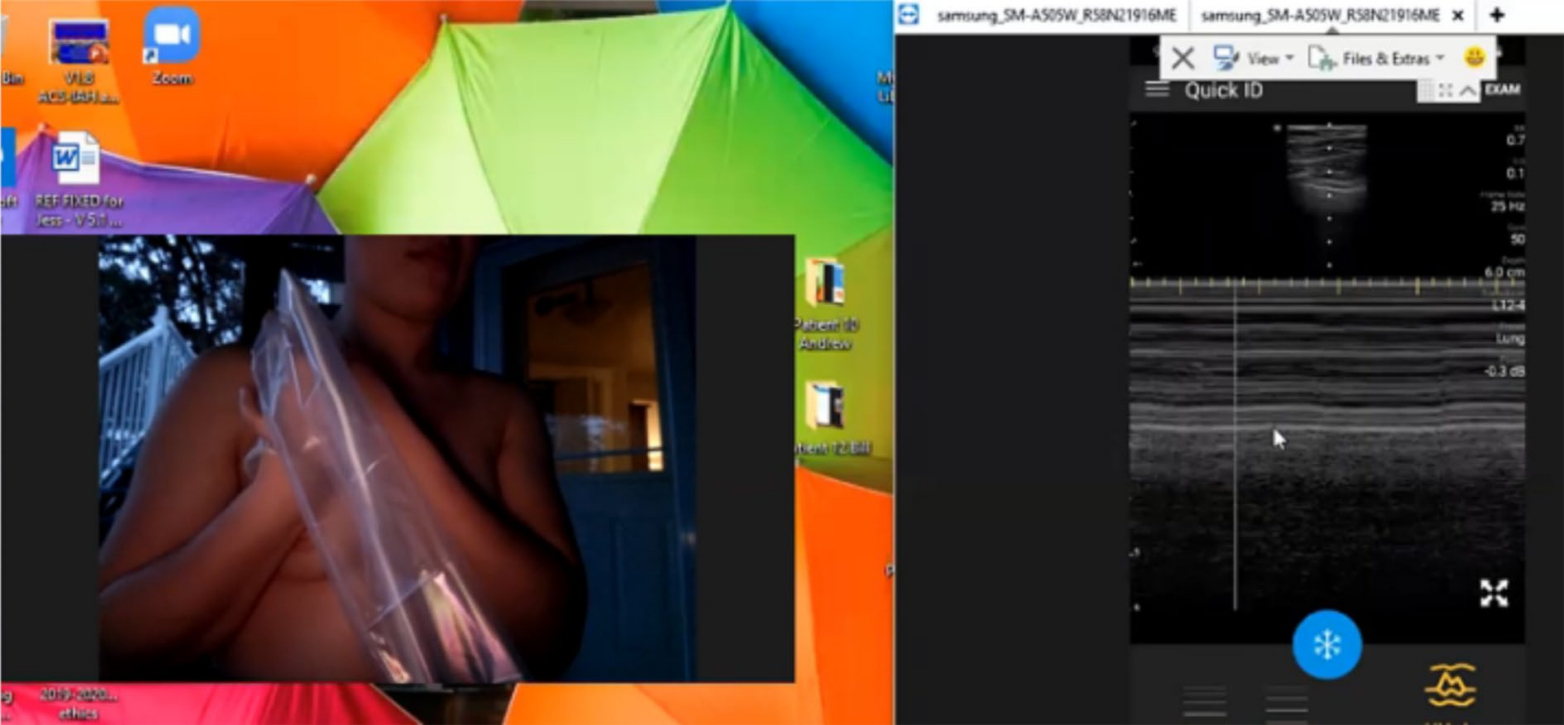
Baseline Self-Performed Lung Ultrasound Study of 31-year-old potentially exposed patient-spouse 6 months prior to patient COVID-19 Diagnosis. Screenshot of the Remote Mentors Computer Screen that shows the patient imaging herself to the left of the screen and the resultant ultrasound image showing the “seashore” sign to the right. As lung ultrasound is a dynamic real-time examination hard to capture in any single image, readers are strongly encouraged to view the accompanying supplemental videos of the entire examination online to gain a true appreciation of the technique

**Fig. 5:**
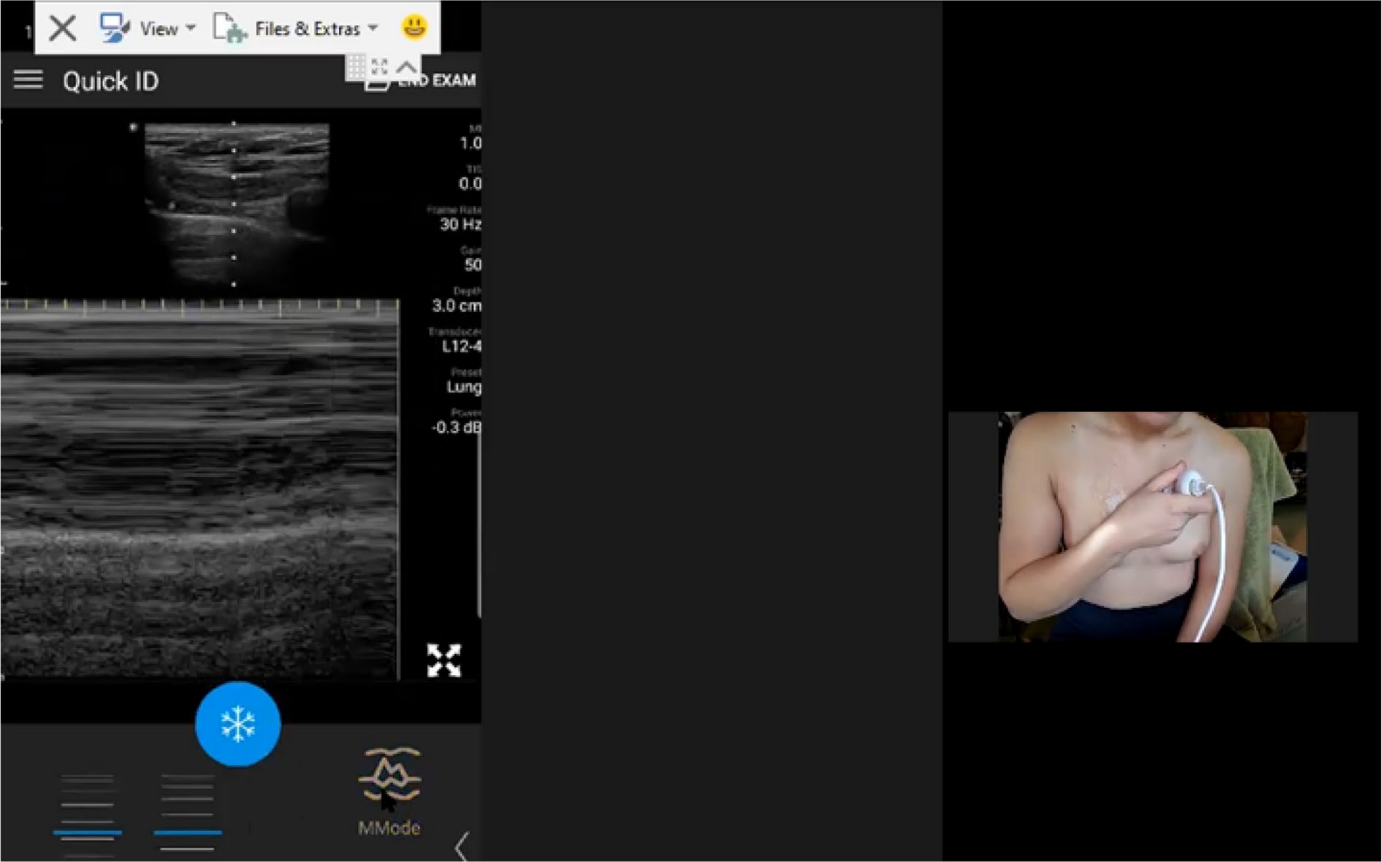
First follow-up Self-Performed Lung Ultrasound Study of 31-year-old potentially exposed patient-spouse 2 weeks after patients COVID-19 Diagnosis. Screenshot of the Remote Mentors Computer Screen that shows the patient imaging herself to the right of the screen and the resultant ultrasound image showing the “seashore” sign to the left. As lung ultrasound is a dynamic real-time examination hard to capture in any single image, readers are strongly encouraged to view the accompanying supplemental videos of the entire examination online to gain a true appreciation of the technique

We are currently conducting a prospective study describing the accuracy and logistics of augmenting RTSPUS to the routine surveillance of paucisymptomatic and at-risk COVID-19 patients. However, the practicality of this approach became dramatically apparent during the recent re-evaluation of our initial self-isolating and healthy volunteers after one of these subjects became infected with COVID-19. Neither subject had ever held an ultrasound prior to agreeing to be remotely mentored. Nevertheless, multiple and complete longitudinal RTSPUS examinations were easily accomplished using widely available cellular phone services, with seamless demonstrations of the pleural anatomy of both the infected patient, and the at-risk partner, during pre- and post-COVID infection examinations (Figs. 1, 2, 3, 4 and 5; Additional files 1, 2, 3, 4 and 5). Fortunately, the infected patient made a complete recovery without requiring hospitalization and testing negative by PCR, and the spouse remained well and PCR negative corresponding to their normal lung ultrasound examinations.

## Potential limitations

It should be understood that at all times this is an additive adjunct to home surveillance which otherwise consists of symptom and standard vital signs monitoring [4]. At all times the responsible supervising clinician may decide to request formal hospital evaluation of a self-isolating patient and the remote lung ultrasound expert is responsible for quality control in interpretation of the self-generated images. Studies have shown, however, that lung ultrasound is superior to either O_2_ saturation or CXR in predicting the future occurrence of adverse outcomes including a requirement for mechanical ventilation and death [17]. The only potential harm envisioned would be if pleural ultrasound findings were falsely interpreted as normal when they were not, but lung ultrasound for COVID has demonstrated an extremely high sensitivity for detecting such abnormalities, which is information that would be unknown if this paradigm was not considered. The high-frequency linear probe utilized optimized the ultrasound images from the pleural interface, but may not be ideal for deeper penetration to detect pathology within the lung or pleural effusions. Other probe selection may optimize this and may warrant further evaluation. The suggested protocol used in this study involved self-scanning posteriorly. Other focused ultrasound examinations for COVID-19 do not require a detailed examination of the back and may be easier for people to be mentored through [17–19]. Only future detailed studies of test-performance characteristics will inform as to whether there is one ideal lung ultrasound examination protocol, but we suspect any and all use of lung ultrasound will benefit patients versus not using this modality. Finally, while the RTSPUS is an example of a method which a completely self-isolating person or one living alone could perform, we also recognize that other self-isolating family members could be mentored to assist or perform the examination on their partners or family as well.

## Future expectations

RTSPUS may potentially revolutionize home-self monitoring for complications of COVID-19. With this paradigm, remote subject matter experts in both infectious diseases and remote tele-ultrasound can easily conference [4, 20], to give at-risk self-isolating patients pleural examinations that will identify those with complicated COVID-19 pneumonia, thus facilitating rapid and early transport to hospital for detailed assessment. Thus, the majority of self-isolating paucisymptomatic infected and asymptomatic at-risk patients can be both better monitored and reassured while completely protecting health-care providers from infection, and reducing the hospital system exposure for these patients to zero until it is deemed necessary that they “enter the hospital”.

## Supplementary Information

**Additional file 1:** Video of baseline self-performed lung ultrasound study of 32-year-old patient 6 months prior to COVID-19 Diagnosis.

**Additional file 2: ** Video of first follow-up self-performed lung ultrasound study of 32-year-old patient 2 weeks after COVID-19 Diagnosis.

**Additional file 3:** Video of second follow-up self-performed lung ultrasound study of 32-year-old patient 2 weeks 2 days after COVID-19 Diagnosis.

**Additional file 4:** Video of baseline self-performed lung ultrasound study of 31-year-old spouse 6 months prior to patients COVID-19 Diagnosis.

**Additional file 5: ** Video of first follow-up self-performed lung ultrasound study of 31-year-old spouse 6 months prior to patients COVID-19 Diagnosis.

## Data Availability

The datasets used and/or analyzed during the current study are available from the corresponding author on reasonable request.

## References

[1] World Health Organization. WHO Coronavirus Disease (COVID-19) Dashboard: World Health Organization; 2020 [updated Dec 6 2020; cited 2020 December 6 2020]. https://covid19.who.int/.

[2] Shahid Z, Kalayanamitra R, McClafferty B, Kepko D, Ramgobin D, Patel R (2020). COVID-19 and older adults: What we know. J Am Geriatr Soc.

[3] Kirkpatrick AW, McKee JL, Volpicelli G, Ma IWY (2020). The potential for remotely mentored patient-performed home self-monitoring for new onset alveolar-interstitial lung disease. Telemed J E Health.

[4] Nacoti M, Ciocca A, Giupponi A, Brambillasca P, Lussana F, Pisano M, et al. At the Epicenter of the Covid-19 Pandemic and Humanitarian Crises in Italy: Changing Perspectives on Preparation and Mitigation. Innovations in Care Delivery. 2020.

[5] Fang Y, Zhang H, Xie J, Lin M, Ying L, Pang P (2020). Sensitivity of chest CT for COVID-19: comparison to RT-PCR. Radiology.

[6] Soldati G, Smargiassi A, Inchingolo R, Buonsenso D, Perrone T, Briganti DF (2020). Proposal for International Standardization of the Use of Lung Ultrasound for Patients With COVID-19: A Simple, Quantitative. Reproducible Method J Ultrasound Med.

[7] Gargani L, Soliman-Aboumarie H, Volpicelli G, Corradi F, Pastore MC, Cameli M (2020). Why, when, and how to use lung ultrasound during the COVID-19 pandemic: enthusiasm and caution. Eur Heart J Cardiovasc Imaging.

[8] Volpicelli G, Gargani L (2020). Sonographic signs and patterns of COVID-19 pneumonia. Ultrasound J.

[9] Volpicelli G, Gargani L, Perlini S, Spinelli S, Barbieri G, Lanotte A (2021). Lung ultrasound for the early diagnosis of COVID-19 pneumonia: an international multicenter study. Intensive Care Med.

[10] Hussain A, Via G, Melniker L, Goffi A, Tavazzi G, Neri L (2020). Multi-organ point-of-care ultrasound for COVID-19 (PoCUS4COVID): international expert consensus. Crit Care.

[11] Kirkpatrick AW, McKee JL (2020). Lung ultrasonography in a woman with COVID-19: This examination could be remote. CMAJ.

[12] Sargsyan AE, Hamilton DR, Jones JA, Melton S, Whitson PA, Kirkpatrick AW (2005). FAST at MACH 20: clinical ultrasound aboard the International Space Station. J Trauma.

[13] Volpicelli G, Elbarbary M, Blaivas M, Lichtenstein DA, Mathis G, Kirkpatrick AW (2012). International evidence-based recommendations for point-of-care lung ultrasound. Intensive Care Med.

[14] Lichtenstein DA, Meziere GA (2008). Relevance of lung ultrasound in the diagnosis of acute respiratory failure: the BLUE protocol. Chest.

[15] Cunningham J, Kirkpatrick AW, Nicolaou S, Liu D, Hamilton DR, Lawless B (2002). Enhanced recognition of "lung sliding" with power color Doppler imaging in the diagnosis of pneumothorax. J Trauma.

[16] Martinelli AW, Ingle T, Newman J, Nadeem I, Jackson K, Lane ND, et al. COVID-19 and pneumothorax: a multicentre retrospective case series. Eur Respir J. 2020. 10.1183/13993003.02697-2020 PMC7487269 32907891

[17] Lichter Y, Topilsky Y, Taieb P, Banai A, Hochstadt A, Merdler I (2020). Lung ultrasound predicts clinical course and outcomes in COVID-19 patients. Intensive Care Med.

[18] Yang Y, Anstey J, Yastrebov K, Nanjayya VB, Orde S, Nalos M (2020). COVID-US: A simplified approach to cardiopulmonary ultrasound in suspected and confirmed COVID-19 patients in surge crisis. Australas J Ultrasound Med.

[19] Lichtenstein DA, Meziere GA (2008). Relevance of lung ultrasound in the diagnosis of acute respiratory distress. Chest.

[20] Netzer I, Kirkpatrick AW, Nissan M, McKee JL, McBeth P, Dobron A (2019). Rubrum Coelis: The Contribution of real-time telementoring in acute trauma scenarios-a randomized controlled trial. Telemed J E Health.

